# Novel Roles of GATA4/6 in the Postnatal Heart Identified through Temporally Controlled, Cardiomyocyte-Specific Gene Inactivation by Adeno-Associated Virus Delivery of Cre Recombinase

**DOI:** 10.1371/journal.pone.0128105

**Published:** 2015-05-29

**Authors:** Terence W. Prendiville, Haidong Guo, Zhiqiang Lin, Pingzhu Zhou, Sean M. Stevens, Aibin He, Nathan VanDusen, Jinghai Chen, Li Zhong, Da-Zhi Wang, Guangping Gao, William T. Pu

**Affiliations:** 1 Department of Cardiology, Boston Children’s Hospital, Boston, Massachusetts, United States of America; 2 Department of Anatomy, School of Basic Medicine, Shanghai University of Traditional Chinese Medicine, Shanghai, China; 3 Institute of Molecular Medicine, Peking-Tsinghua Center for Life Sciences, Peking University, Beijing, China; 4 Gene Therapy Center, University of Massachusetts Medical School, Worcester, Massachusetts, United States of America; 5 Harvard Stem Cell Institute, 1350 Massachusetts Ave, Cambridge, Massachusetts, United States of America; The University of Queensland, AUSTRALIA

## Abstract

GATA4 and GATA6 are central cardiac transcriptional regulators. The postnatal, stage-specific function of the cardiac transcription factors GATA4 and GATA6 have not been evaluated. In part, this is because current Cre-loxP approaches to cardiac gene inactivation require time consuming and costly breeding of Cre-expressing and “floxed” mouse lines, often with limited control of the extent or timing of gene inactivation. We investigated the stage-specific functions of GATA4 and GATA6 in the postnatal heart by using adeno-associated virus serotype 9 to control the timing and extent of gene inactivation by Cre. Systemic delivery of recombinant, adeno-associated virus 9 (AAV9) expressing Cre from the cardiac specific *Tnnt2* promoter was well tolerated and selectively and efficiently recombined floxed target genes in cardiomyocytes. AAV9:Tnnt2-Cre efficiently inactivated *Gata4* and *Gata6*. Neonatal *Gata4/6* inactivation caused severe, rapidly lethal systolic heart failure. In contrast, *Gata4/6* inactivation in adult heart caused only mild systolic dysfunction but severe diastolic dysfunction. Reducing the dose of AAV9:Tnnt2-Cre generated mosaics in which scattered cardiomyocytes lacked *Gata4/6*. This mosaic knockout revealed that *Gata4/6* are required cell autonomously for physiological cardiomyocyte growth. Our results define novel roles of GATA4 and GATA6 in the neonatal and adult heart. Furthermore, our data demonstrate that evaluation of gene function hinges on controlling the timing and extent of gene inactivation. AAV9:Tnnt2-Cre is a powerful tool for controlling these parameters.

## Introduction

GATA4 and GATA6 are critical cardiac transcription factors with partial functional redundancy [1,2]. Previous gene inactivation studies have interrogated the function of GATA4 and GATA6 using several different cardiac Cre alleles. Inactivation in cardiac progenitors or early cardiomyocytes using Nkx2-5^Cre^ showed that GATA4 is required for cardiomyocyte proliferation and embryonic survival [3]. Later fetal inactivation of either GATA4 or GATA6 with Myh6-Cre (also known as MHCα-Cre) permitted embryonic survival [4,5]. These mutant mice developed progressive dilated cardiomyopathy with severe systolic dysfunction and died in adulthood. Furthermore, these mice had attenuated cardiomyocyte hypertrophy in response to pressure overload, indicating that GATA4 and GATA6 are required for this pathological growth response. Surprisingly, inactivation of both *Gata4* and *Gata6* with Myh6-Cre was compatible with embryonic survival, and suggested an additive effect of combined inactivation of both GATA4 and GATA6 [2,4]. However, the extent to which the adult phenotypes reflect developmental roles of GATA4 or GATA6 or their function in the adult heart remain uncertain.

Genetic loss of function is a commonly used strategy to decipher essential gene functions in vivo. In cardiac biology, floxed genes are inactivated most commonly by constitutive Cre alleles active in the fetal heart. While this strategy has been highly productive, it can lead to experimental hurdles (e.g., inability to analyze gene function due to premature death). Moreover, the interpretation of experiments can be confounded by the impact of developmental changes on later stage phenotypes, and by secondary consequences of organ-wide gene inactivation. Recently, “inducible Cre”-based genetic inactivation strategies have been increasingly used to achieve temporal control of gene inactivation. Most often this is achieved using Cre fused to tamoxifen-activated variants of the estrogen hormone binding domain, such as CreERT2 or MerCreMer [6,7]. However, incomplete gene inactivation and toxicity related to the combination of these fusion genes and tamoxifen, including cardiac fibrosis and left ventricular dysfunction, are additional difficulties that must be addressed when using these inducible Cre experimental models [8–11].

Here, we use adeno-associated virus (AAV) as an alternative approach to achieve control the timing and extent of *Gata4* and *Gata6* cardiac inactivation. AAV is a highly efficient, non-pathogenic cardiac gene transfer vehicle that elicits little significant immune response [12]. AAV serotype 9 (AAV9) efficiently delivers its cargo to post-mitotic cells including cardiomyocytes [13], and we show that AAV9-mediated Cre delivery is a simple and effective means to control the timing and extent of cardiomyocyte gene inactivation. By controlling the extent and timing of gene inactivation with AAV9 delivery of Cre, we uncovered novel roles of *Gata4* and *Gata6* in the growth, contractile maturation, and diastolic function of the postnatal heart. These phenotypes differ from those that result from fetal knockout of *Gata4/6 [4]*, highlighting how the timing and extent of gene inactivation critically impact the interpretation of experimental results.

## Materials and Methods

### Adeno-associated Virus

AAV9:Tnnt2-Cre and AAV:Tnnt2-Luc were constructed by placing codon-optimized Cre [14] or Luciferase into an ITR-containing AAV plasmid (Penn Vector Core P1967) harboring the chicken cardiac TNT promoter. AAV constructs were propagated in Stbl2 competent cells (Life Technologies; Cat. # 10268–019), and their integrity was validated by restriction digestion and DNA sequencing. AAV9 was packaged at the Viral Vector Core of the Gene Therapy Center, University of Massachusetts Medical School. Viral titer was determined by quantitative PCR. AAV:Tnnt2-Cre contained IRES-TdTomato, although the level of TdTomato expression was not detectable by microscopy. Aliquots were stored at -80°C prior to use.

### Mice

All experiments involving animals were approved by the Boston Children’s Hospital Animal Care and Use Committee. *Gata4 [15]*, *Gata6* (JAX# 008196) [16], and *Rosa26* ^*mTmG/mTmG*^ [17] mice were obtained from Jackson Labs. Mice were injected under light isoflurane anesthesia at a dose of 1x10^11^ VP/gram body weight by injection (pups). Pups and adults were injected intraperitoneally and intravascularly, respectively.

### Physiological measurements

Echocardiography was performed under light anesthesia (0.5%-1.5% isoflurane mixed with oxygen) on a heated animal platform using the Vevo 2100 imaging system with MS550D (40MHz) transducer (VisualSonics Inc.). Mice were monitored for core temperature, heart rate and level of anesthesia. Ventricular volumes, wall thickness and function (ejection fraction or shortening fraction) were determined from M-mode cine loops taken in the parasternal short and long axis planes. B-mode cine loops were also acquired and analyzed using Vevostrain analysis software (VisualSonics Inc.). Mitral valve inflow gradient was acquired using Doppler interrogation at the level of the valve annulus from a 4-chamber view.

Closed chest cardiac catheterization was performed under light anesthesia (1–3% isoflurane mixed with oxygen) using a 1.2F SciSense ADVantage pressure catheter and accompanying system for measuring pressure-volume loops. Access was obtained through cannulation of the right carotid artery under local dissection, and the catheter was advanced across the aortic valve into the left ventricular cavity. Further data analysis was done using Labchart v.8 software.

### Histology

Myocardial cryostat sections (10 μm) were immunostained using antibodies listed in Table 1. Sections were mounted with Vectashield with DAPI and imaged using an Olympus FV1000 confocal microscope. Other sections were stained with picro-sirius red and fast green and imaged with a Nikon SMZ1000 dissecting microscope.

**Table 1:** Antibodies used in this study.

Gene	Vendor	Cat No	Dilution	Purpose
GATA4	Santa Cruz	Sc-9053	1:1,000	WB
GATA6	Cell Signaling	4253S	1:1,000	WB
GAPDH	Fitzgerald Ind.	10R-G109a	1:20,000	WB
TNNI3	Abcam	Ab56357	1:250	IF

WB, western blot; IF, immunofluorescent staining.

### Gene expression

Western blot analysis of mouse neonatal heart homogenates was performed using antibodies indicated in Table 1.

For quantitation of RNA expression, total RNA was extracted from snap-frozen ventricular myocardial tissue. RNA was purified using an RNeasy Mini kit (Qiagen, Cat. # 74104). cDNA was made from total RNA using Superscript III first-strand synthesis system (Invitrogen; Cat. # 18080–051). qPCR was performed on a Bio-Rad CFX96 real-time system using Fast SYBR Green Master mix (Applied Biosystems, Cat. # 4385612) using primers indicated in Table 2.

**Table 2:** Primers used in this study.

Gene	Sequence
Gata4-F	GCCGAGGGAGCCGCCTACAC
Gata4-R	TGGGGTGTCTTCCAGGGTTGG
Gata6-F	AGTTTTCCGGCAGAGCAGTA
Gata6-R	GCACTTGGAGCTGTAGGTCA
Myh7-F	AAGGGCCTGAATGAGGAGTAGATC
Myh7-R	TGCAAAGGCTCCAGGTCTGA
Myh6-F	ACATGAAGGAGGAGTTTGGG
Myh6-R	GCACTTGGAGCTGTAGGTCA
Nppa-F	GGCCATATTGGAGCAAATCCTGTG
Nppa-R	CATGACCTCATCTTCTACCGGCAT
GAPDH	Life Technologies Taqman assay #4352932E

Primers used in this study are indicated (5’-3’).

### Cardiomyocyte dissociation

Neonatal hearts were dissociated using the Miltenyi neonatal heart dissociation kit, and cardiomyocytes were purified by magnetic cell sorting (Miltenyi Biotec #130-098-373). Fluorescence-activated cell sorting (FACS) was performed on a BD FACSAria II. Adult hearts were dissociated by retrograde collagenase perfusion [18].

### Cell size measurement

In cryosections, cell cross-sectional area was measured in ImageJ by manually outlining cardiomyocytes based on the membrane-localized GFP or RFP signal. Adult hearts were dissociated by retrograde collagenase perfusion and cardiomyocytes were isolated by differential centrifugation as described [18]. Random fields of cardiomyocytes were imaged and the projected area was measured by manually outlining cardiomyocytes in ImageJ. Cells were grouped into those with and without GFP expression.

### Statistics

All results are presented as mean ± SEM. Statistical analyses were performed in Microsoft Excel and GraphPad Prism (v6) using an unpaired t-test (qPCR and echocardiography data) and the Mann-Whitney U test (cardiac catheterization and cell size data). A p-value of < 0.05 was considered significant.

## Results

### Cardiomyocyte-specific recombination of floxed genes by AAV9-Tnnt2-Cre

**Fig 1:**
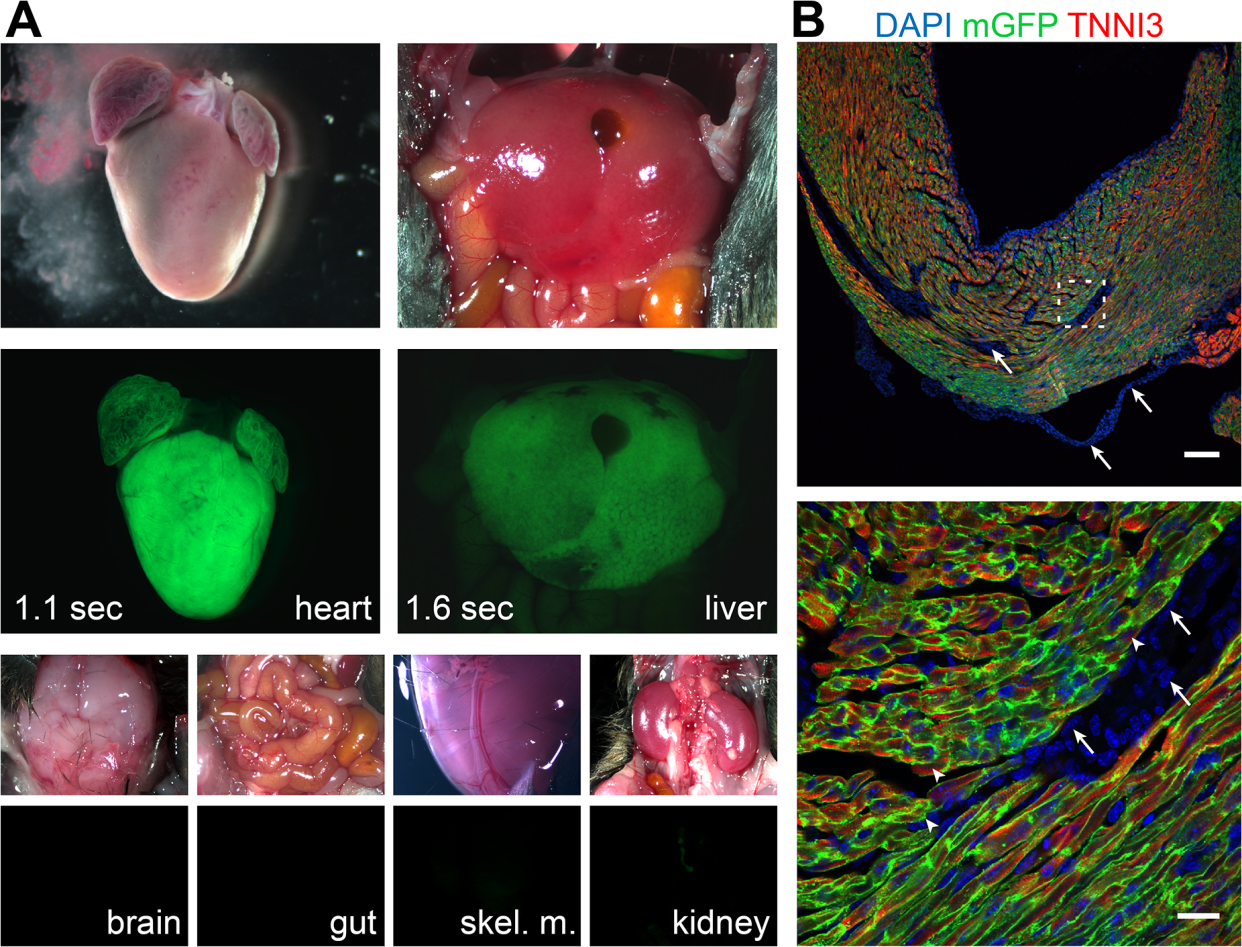
Cardiac-selective gene inactivation with AAV9-Tnnt2-Cre. AAV9-Tnnt2-Cre was administered to Rosa26^mTmG^ neonatal mice. Cre recombination activates GFP expression. **(A)**. Brightfield and GFP fluorescent signal from the indicated organs. skel. m., skeletal muscle. **(B)**. Immunofluorescent staining of heart cryosections. Green indicates native membrane-localized GFP fluorescence. Cardiomyocytes were stained by TNNI3 (representative examples indicated by arrowheads). Boxed area in top image is magnified in the bottom image. Arrows indicate non-myocytes. Bar, 100 μm (C, top) and 20 μm (C, bottom).

We generated AAV9 containing the cardiomyocyte-selective Tnnt2 promoter driving mammalian codon optimized Cre (AAV9:Tnnt2-Cre). We intraperitoneally injected the virus into neonatal Rosa26^mTmG^ Cre-reporter mice, in which Cre recombination activates membrane-localized green fluorescent protein (mGFP) and inactivates red fluorescent protein (mRFP). Systemically delivered AAV9:Tnnt2-Cre selectively activated mGFP in the heart (Fig 1A). Weak GFP fluorescence was detected in the liver, and no GFP was detected in other organs. Within the heart, we observed robust activation of mGFP in cardiomyocytes (Fig 1B). Cell counting showed that 95.3 ± 1.3% of cardiomyocytes expressed mGFP (n = 3). We did not detect expression of mGFP in non-cardiomyocytes (arrows, Fig 1B).

**Fig 2:**
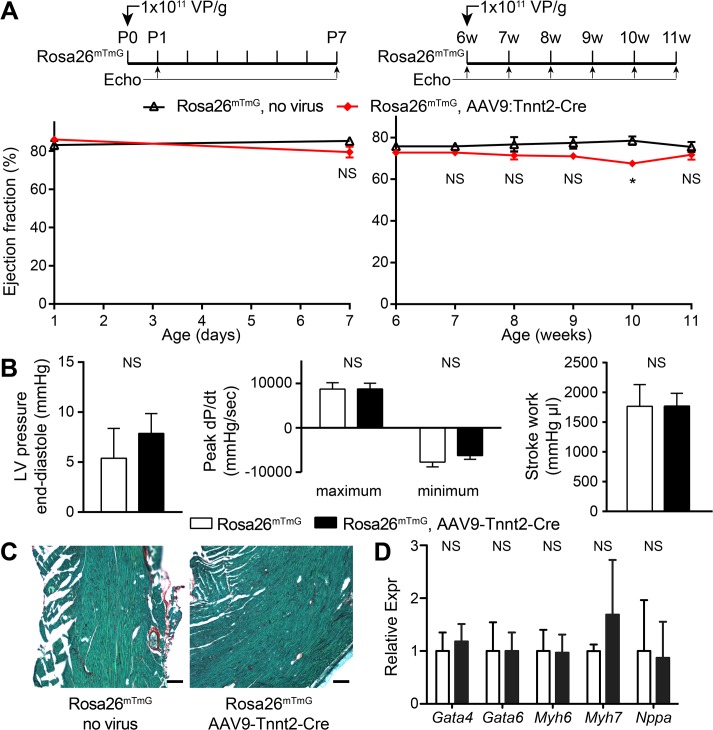
AAV9:Tnnt2-Cre had minimal effect on Rosa26^mTmG^ heart functional parameters. **(A)**. Neonatal (left) and adult (right) treatment timelines and echocardiographic evaluation of heart function. n = 8 per group. *, P<0.05. **(B)**. Closed chest invasive hemodynamics assessment of effect of AAV9:Tnnt2-Cre on cardiac function. n = 5 per group. **(C)**. Fast green-picrosirius red staining of heart sections. Red staining indicates fibrosis. Bar = 30 μm. **(D)**. qRTPCR assessment of relative gene expression. n = 3. NS, not significant.

AAV9:Tnnt2-Cre was well tolerated in the normal heart. It had little detectable effect on normal heart function by either echocardiography or invasive hemodynamics (Fig 2A and 2B), did not induce fibrosis (Fig 2C), and did not alter expression of *Myh6* (also known as MHCα), cardiac transcription factors *Gata4* and *Gata6*, or cardiac stress markers *Nppa* (also known as ANP) and *Myh7* (also known as MHCβ; Fig 2D).

### Neonatal inactivation of GATA4/6 causes rapidly lethal systolic heart failure

**Fig 3:**
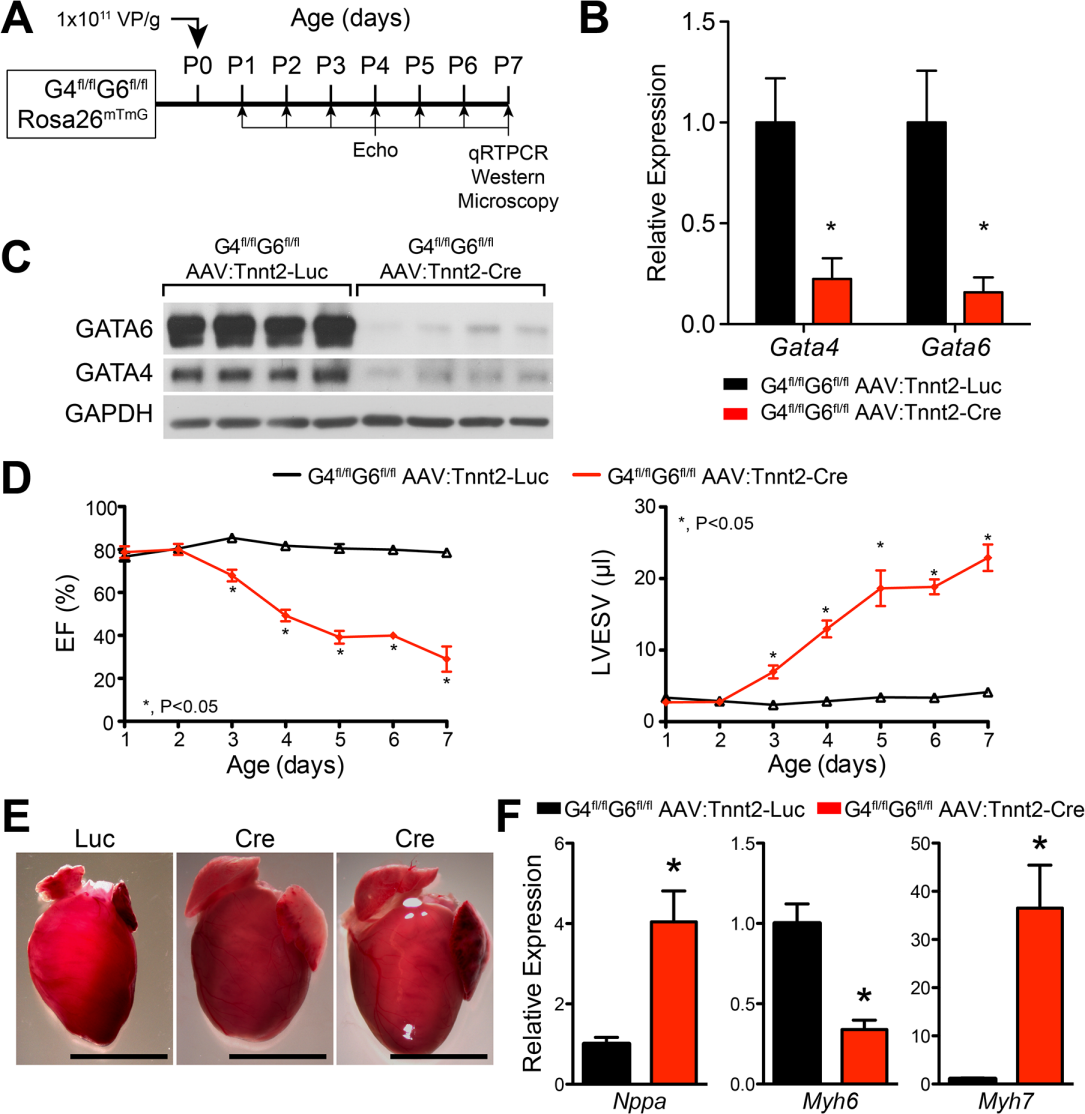
AAV9:Tnnt2-Cre mediated inactivation of *Gata4/6* in neonatal heart. **(A)**. Experimental timeline. AAV9:Tnnt2-Cre or AAV9:Tnnt2-Luc (control) was administered to P0 pups. Mice were studied daily by echocardiography and tissues were analyzed at P7. **(B)**. qRTPCR assessment of *Gata4* and *Gata6* inactivation in myocardium. **(C)**. Immunoblotting assessment of GATA4 and GATA6 inactivation in myocardium. **(D)**. LV systolic function, measured by echocardiography. EF, ejection fraction. LVESV, left ventricular end systolic volume. n = 7 per group. *, P<0.05. **(E)**. Gross morphology. Bar = 5 mm. **(F)**. Expression of *Nppa*, *Myh6*, and *Myh7*, quantified by qRTPCR at P7. n = 3. *, P<0.05.

We used AAV9:Tnnt2-Cre to inactivate floxed alleles of GATA4 and GATA6 in cardiomyocytes. *Gata4* and *Gata6* are critical cardiac transcription factors with partial functional redundancy in fetal heart development [1]. We treated neonatal GATA4^fl/fl^::Gata6^fl/fl^::Rosa26^mTmG^ mice with AAV9:Tnnt2-Cre (Fig 3A). AAV9:Tnnt2-Cre rapidly inactivated both *Gata4* and *Gata6*, as both mRNA and protein were dramatically down-regulated by 5–7 days after virus administration (Fig 3B and 3C). Control neonates injected with AAV9:Tnnt2-Luc at P1 retained normal ventricular function and experienced no mortality. In contrast, AAV9:Tnnt2-Cre treatment caused profound, rapidly progressive systolic dysfunction, ventricular dilatation, and death by 7 days post-injection (Fig 3D and 3E; S1 Table). Rapid death of these mice was surprising given that mice with fetal *Gata4/6* inactivation by Myh7-Cre (also known as MHCβ-Cre) survived normally through the neonatal period and into adulthood [4], when they died by ~100 days of age from dilated cardiomyopathy.

Cardiac stress induces a set of genes to revert towards their fetal expression pattern [19,20]. Among these are upregulated genes *Nppa* and *Myh7*, and downregulated gene *Myh6*. GATA4 has been implicated in the activation of these genes [21–23]. Therefore we investigated the effect of neonatal *Gata4/6* inactivation on expression of these genes. We found that neonatal *Gata4/6* inactivation upregulated both *Nppa* and *Myh7* and downregulated *Myh6* (Fig 3F). Upregulation of *Nppa* and *Myh7* despite loss of GATA4/6 indicates that their hypertrophy-driven activation does not require GATA4/6.

### Adult GATA4/6 inactivation causes lethal diastolic heart failure

**Fig 4:**
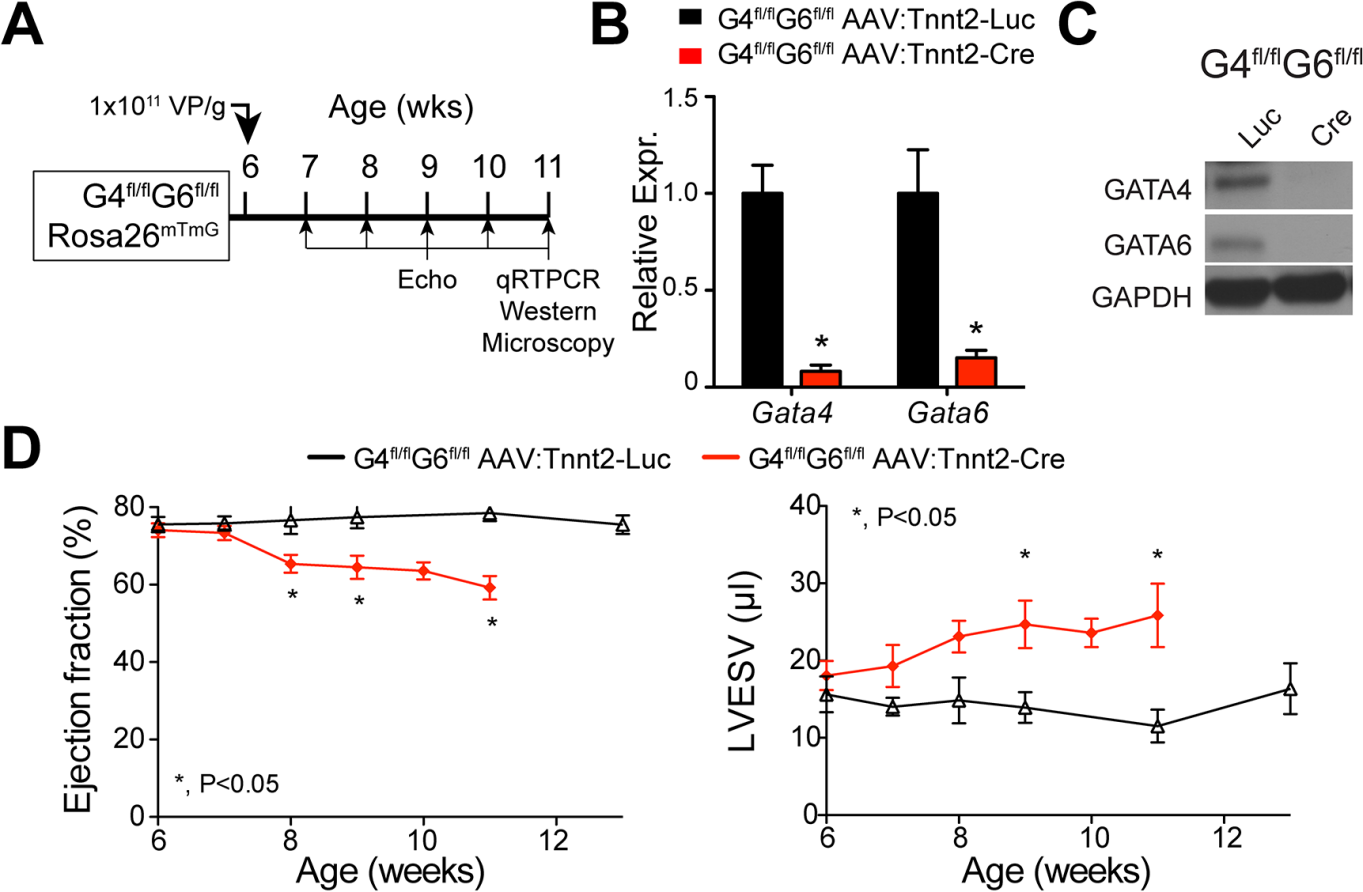
AAV9:Tnnt2-Cre inactivation of *Gata4/6* in adult heart. **(A).** Experimental timeline. Virus was administred at 6 weeks of age. After weekly echocardiography, tissue was analyzed at 11 weeks of age. **(B).** Quantitation of *Gata4* and *Gata6* inactivation in ventricular myocardium by qRTPCR. **(C).** Loss of GATA4 and GATA6 protein with AAV9:Tnnt-Cre but not AAV9:Tnnt2-Luc. Ventricular myocardial protein was analyzed by western blotting. **(D).** Mild systolic dysfunction following adult stage inactivation of Gata4 and Gata6, as determined by echocardiography. n = 9 (knockout) or 4 (control). *, P<0.05.

**Fig 5:**
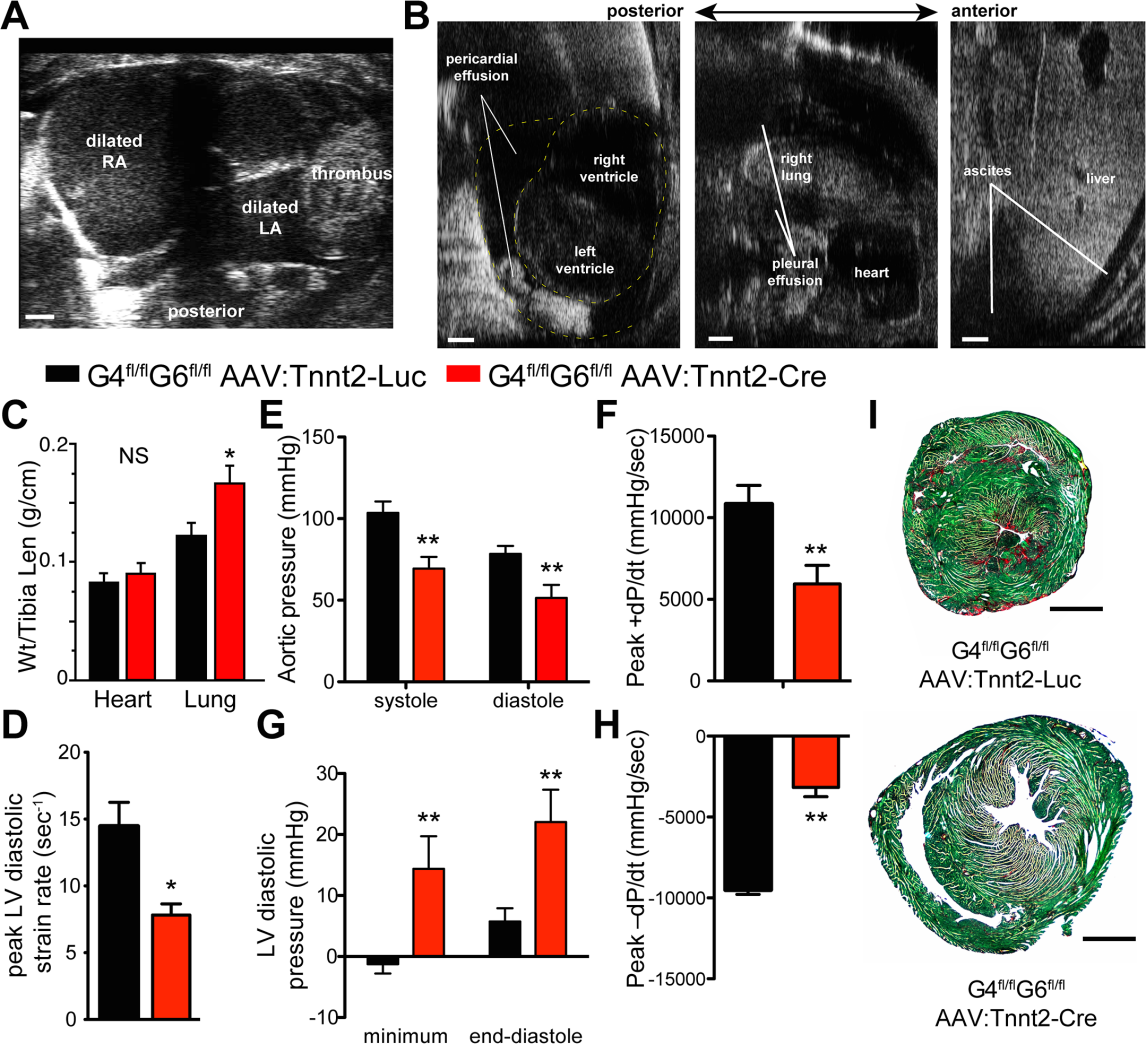
Diastolic dysfunction following Gata4/6 inactivation in adult heart. **Studies were performed 5 weeks after AAV delivery. (A-B).** Echocardiographic images were obtained Images show bi-atrial dilatation and intra-atrial thrombus (A) and pericardial, pleural, and abdominal fluid collections (B). Bar = 1 mm. **(C).** Heart and lung weights, normalized to tibia length. NS, not significant. *, P<0.05. n = 5 Luc, 7 Cre). **(D).** Echocardiographic assessment of diastolic function by peak diastolic strain rate. **, P<0.01. n = 4 Luc, 9 Cre. **(E-H)**. Closed chest invasive hemodynamic assessment of left ventricular systolic (E,F) and diastolic (G,H) function. n = 5 per group. **, P<0.01. **(I)**. Fast green picrosirius red assessment of ventricular fibrosis. No significant difference was observed between control and GATA4/6 mutant hearts. Bar = 500 μm.

Given the difference in phenotype between fetal and neonatal *Gata4/6* inactivation, we asked if adult inactivation of these genes would also cause a distinct phenotype. We administered AAV9:Tnnt2-Cre or AAV9:Tnnt2-Luc intravascularly to 6-week old Gata4^fl/fl^::Gata6^fl/fl^::Rosa26^mTmG^ mice (Fig 4A). By 11 weeks of age, cardiac *Gata4* and *Gata6* transcripts were less than 10% of control levels (Fig 4B), and western blotting confirmed loss of GATA4 and GATA6 proteins (Fig 4C). Echocardiography showed that the LV developed mild dysfunction (EF>60%) and dilatation (Fig 4D and S2 Table). Despite these mild changes in systolic function and ventricular volume, Cre-treated mice exhibited disproportionate signs and symptoms of heart failure and required euthanasia by six weeks of age for humanitarian reasons. Echocardiography revealed pronounced bi-atrial dilation (Fig 5A), ascites, and pleural and pericardial effusions (Fig 5B). These abnormalities were not due to possible *Gata4*/6 inactivation in liver, since hepatic inactivation of both genes in adults did not cause a significant phenotype [24]. Pulmonary congestion was confirmed by gravimetric analysis of the lungs (Fig 5C), while heart weight was not significantly changed, consistent with the mild cardiac dilatation.

As a result of the overt signs of heart failure and atrial dilation despite relatively preserved LV systolic function up to the time of death, we hypothesized that diastolic dysfunction was the underlying hemodynamic mechanism. Consistent with diastolic dysfunction, speckle-tracking echocardiography showed a significant decline in peak LV diastolic strain (p = 0.028; Fig 5D). Diastolic dysfunction was confirmed by invasive hemodynamics (Fig 5E–5H). AAV9:Tnnt2-Cre-treated mice had lower aortic pressure and reduced maximal +dP/dt, consistent with slightly reduced systolic function (Fig 5E and 5F). There was a profound elevation in LV end-diastolic pressure and blunted minimum peak -dP/dt, both markers of diastolic dysfunction (Fig 5G and 5H). It is unlikely that fluid accumulations accounted for the observed hemodynamic derangements, since echocardiography of the catheterized mice showed that only two of five had ascites and pericardial effusions, and the severity of the hemodynamic abnormalities did not correlate with presence of effusions. In summary, the hemodynamic analysis showed that adult cardiomyocyte inactivation of *Gata4/6* cardiomyocyte depressed diastolic function while only mildly impairing systolic function.

Diastolic dysfunction can be caused by myocardial fibrosis. Picro-sirius red/fast green staining of adult GATA4/6 knockouts did not reveal a substantial increase in fibrosis (Fig 5I). Thus impaired diastolic function of *Gata4/6* knockout hearts was likely caused by abnormal cardiomyocyte relaxation.

**Fig 6:**
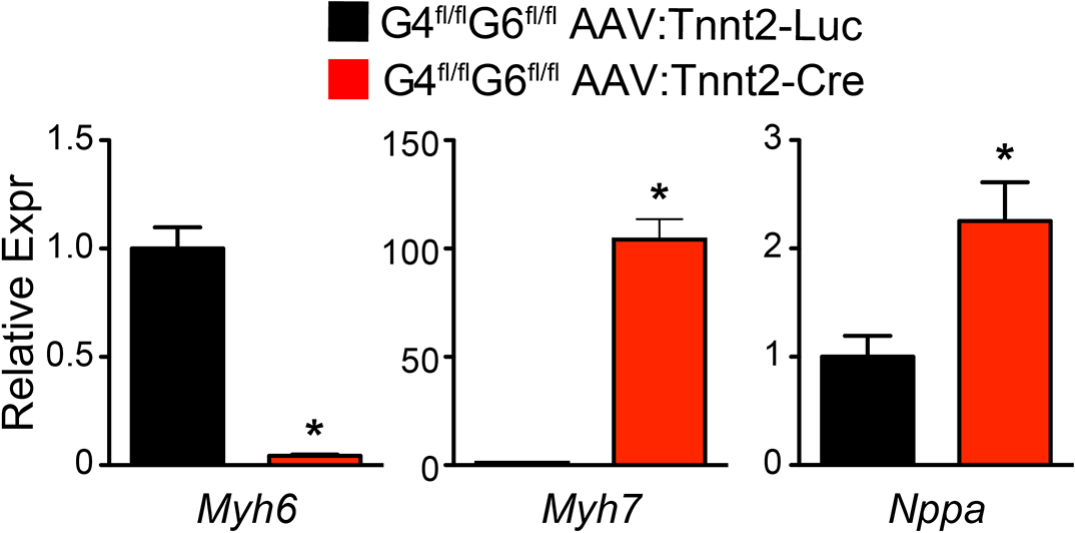
Expression of cardiac stress markers after adult cardiac inactivation of *Gata4* and *Gata6*. qRTPCR was performed on ventricular myocardial RNA. n = 6. *, P<0.05.

We measured changes in gene expression in the *Gata4/6* adult stage knockouts (Fig 6). As in the neonatal *Gata4/6* loss-of-function experiment, *Myh7* and *Nppa* were highly upregulated in GATA4/6 knockout hearts, while *Myh6* was highly downregulated, consistent with changes observed in heart failure.

### Mosaic Gata4/6 inactivation identifies cell autonomous roles

Organ-wide cardiomyocyte gene inactivation causes both primary changes directly resulting from gene inactivation, as well as secondary changes associated with organ dysfunction. In some cases, such as neonatal GATA4/6 knockout by AAV9:Tnnt2-Cre, death complicates study of gene function in processes such as physiological growth. These limitations could be overcome by mosaic gene inactivation in a fraction of cells.

**Fig 7:**
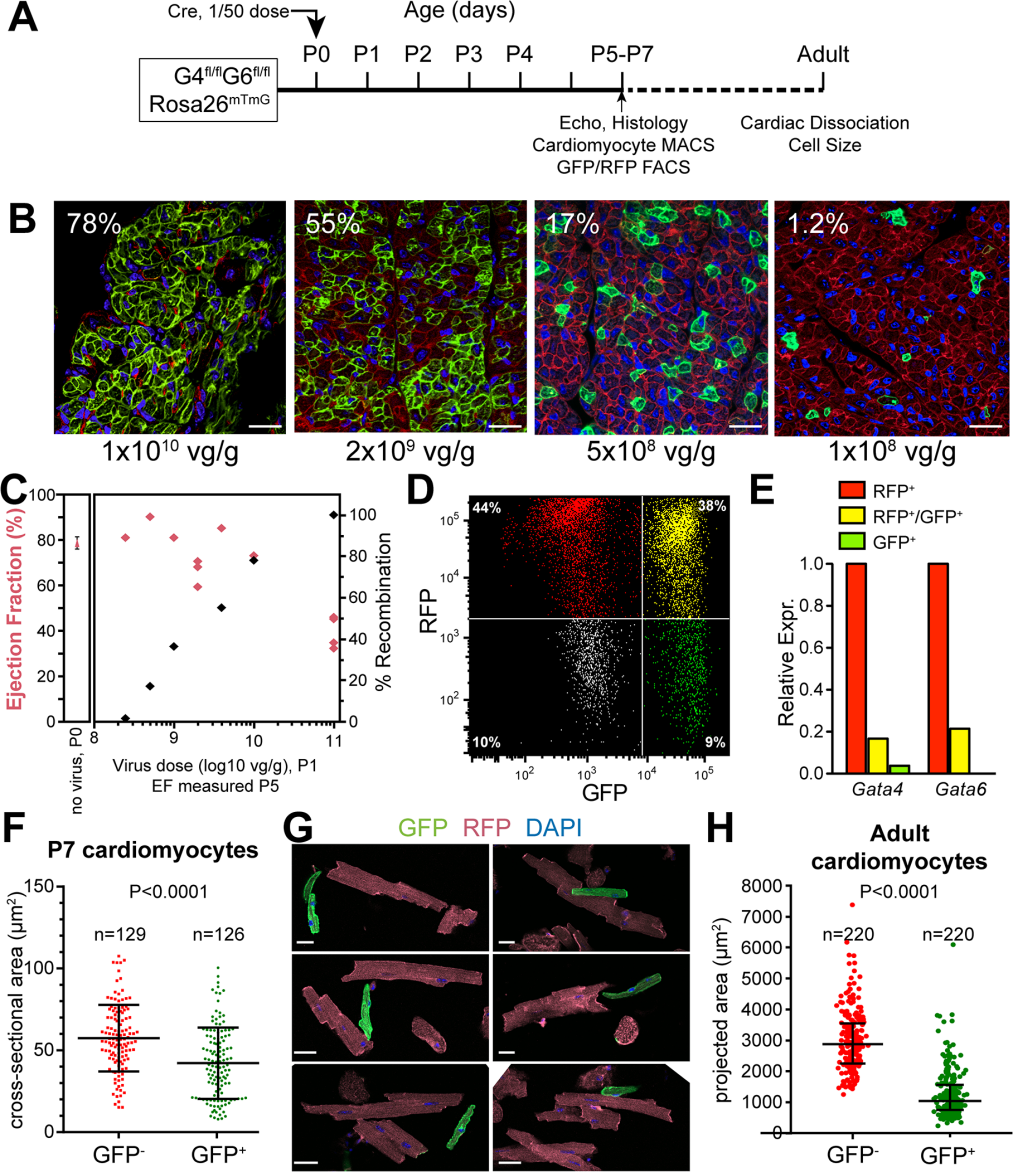
Mosaic inactivation of *Gata4/6* in the neonatal heart. **(A).** Experimental timeline. **(B).** Titration of AAV9:Tnnt2-Cre in neonatal Rosa26^mTmG^ mice. GFP marks Cre-recombined cells. The percentage of GFP^+^ cells is indicated. Bar = 20 μm. **(C).** Relationship of virus dose to ejection fraction (red, left axis) and recombination efficiency (black, right axis). **(D-E).** Gata4^fl/fl^::Gata6^fl/fl^::Rosa26^mTmG^ neonates were treated with low dose AAV9:Tnnt2-Cre. Cardiomyocytes were purified at P7 by magnetic cell sorting, then FACS sorted (D). *Gata4* and *Gata6* levels, measured by qRTPCR, confirmed gene knockout in GFP^+^ cells (E). **(F).** Size of GATA4/6 knockout cardiomyocytes at P7. The cross sectional area of GFP^+^ (recombined) and RFP^+^ (non-recombined) cardiomyocytes in P7 cryosections was compared using the Mann-Whitney test. **(G-H).** Size of GATA4/6 deficient cardiomyocytes in adult mosaic knockout hearts. Representative images of cardiomyocytes dissociated from adult hearts with mosaic GATA4/6 knockout by AAV9:Tnnt-Cre administered at P1. (G). Bar = 20 μm. Quantitation of GFP^+^ and GFP^-^ cardiomyocyte size (H) showed that GFP^+^ cardiomyocytes were significantly smaller by Mann-Whitney test. In F and H, black lines represent the median and 1st and 3rd quartiles.

We used AAV9-Tnnt2-Cre to induce mosaic inactivation of GATA4/6 in the neonatal heart. We administered serial dilutions of AAV9:Tnnt2-Cre to P1 GATA4^fl/fl^::GATA6^fl/fl^::Rosa26^mTmG/mTmG^ mice and observed progressively lower frequency of recombined cells (Fig 7A and 7B). 2x10^9^ viral genomes/g body weight (i.e., 1:50 of the standard dose) did not globally affect heart systolic function (Fig 7C) but still yielded enough GFP^+^ cells for downstream analysis. We used this dose for subsequent mosaic inactivation studies. We isolated the GFP^+^, RFP^+^, and GFP^+^RFP^+^ cardiomyocyte populations from dissociated P7 hearts (Fig 7D). qRTPCR showed that GATA4 and GATA6 were reduced by over 80% in GFP^+^ and GFP^+^RFP^+^ cells compared to RFP^+^ cells (Fig 7E). Thus GFP marks GATA4/6 deficient cells.

Rapid systolic dysfunction following newborn cardiac GATA4/6 inactivation (Fig 3) led us to hypothesize that GATA factors are required for neonatal cardiomyocyte maturation, a hallmark of which is physiological cardiomyocyte hypertrophy. To determine the cell autonomous requirement of GATA4/6 for neonatal cardiomyocyte growth, we delivered diluted AAV9:Tnnt2-Cre to P1 GATA4^fl/fl^::GATA6^fl/fl^::Rosa26^mTmG/mTmG^ mice. In histological sections at p7, cardiomyocyte cross-sectional area of knockout cardiomyocytes (GFP^+^) was smaller than controls (RFP^+^; 0.73-fold, P<0.0001 by Mann-Whitney; Fig 7F). The mice survived to adulthood with normal heart function, permitting us to assess cell autonomous GATA4/6 function for the entire period of normal physiological heart growth. Dissociation of mosaic knockout adult hearts revealed that GATA4/6 knockout cardiomyocytes were much smaller than controls (P<0.0001 by Mann-Whitney; Fig 7G and 7H). These results indicate that GATA4/6 are required for normal postnatal heart growth.

## Discussion

By inactivating *Gata4* and *Gata6* in cardiomyocytes specifically in the neonatal or adult heart, we uncovered novel stage-specific roles of these genes. Acute loss of *Gata4* and *Gata6* in the perinatal period caused rapid loss of systolic function. This is a critical period in heart development, during which time cardiomyocytes adapt to substantially higher hemodynamic demands by undergoing numerous changes in their size, ultrastructure, sarcomere composition, and metabolism. Lack of cardiomyocyte maturation is currently a major bottleneck to using reprogrammed or stem cell-derived cardiomyocytes for cardiac repair and regeneneration [25]. Our data show that perinatal cardiomyocyte inactivation of *Gata4/6* dramatically impaired neonatal systolic function, and mosaic inactivation of these genes revealed their essential roles in physiological postnatal cardiomyocyte growth. Together, these data indicate that *Gata4/6* are key regulators of postnatal cardiomyocyte maturation. Further mechanistic studies of this knockout model will illuminate critical steps in the maturation process and its regulation by *Gata4* and *Gata6*.

Inactivation of *Gata4* and *Gata6* in adult cardiomyocytes uncovered an unexpected requirement of these genes to regulate mature heart diastolic function. This diagnosis was supported by signs of severe heart failure in the face of only mild systolic dysfunction, by atrial dilatation out of proportion to ventricular dilatation, and by echocardiographic and invasive hemodynamic measures of diastolic function. Diastolic heart failure is a significant cause of heart failure in adult patients [26], but far less is known about the factors that govern diastolic heart function than systolic heart function. The adult *Gata4/6* knockout mouse will provide a fruitful model to investigate the pathophysiology of diastolic heart failure.

Combined inactivation of *Gata4* and *Gata6* by Myh7-Cre in fetal development was reported previously [4]. Fetal knockout of these genes was compatible with postnatal survival for up to 100 days. Surprisingly, our work shows that acute neonatal knockout was quite different and resulted in death within 7 days. The difference between chronic and acute loss of function may be due to secondary, compensatory adaptations that occur in the chronic model. We hypothesize that such compensatory changes masked the essential role of *Gata4* and *Gata6* in neonatal cardiomyocyte maturation and in maintenance of normal adult heart diastolic function. Similar compensatory changes have likewise been proposed to account for differences between acute miRNA silencing with antagomirs, compared to their chronic silencing in genetic models [27].

The Gata4^fl/fl^::Gata6^fl/fl^::Myh7-Cre mouse model was interpreted to show an essential role of these transcription factors in regulating adult heart systolic function; however, our data on inactivation of these genes in the adult heart suggest that the systolic heart failure phenotype was a result of abnormal heart development in the absence of these key transcriptional regulators. Moreover, this developmental phenotype masked a bone fide function of GATA4/6 in the adult heart, the regulation of adult heart diastolic function. These results point out the critical importance of regulating the timing of gene inactivation to gain insights into gene function most relevant to the question under investigation. It is likely that many other genes that have been studied in the adult heart following inactivation by developmentally active Cre, such as Myh6-Cre or Myh7-Cre, should be reassessed by temporally controlled gene inactivation in the adult heart.

The stage-specific functions of Gata4/6 in fetal, newborn, and adult stages likely reflect their regulation of stage-specific gene programs. Consistent with this, we recently demonstrated that GATA4 genome occupancy is highly dynamic, with most GATA4 binding sites changing between the fetal and adult heart [28]. Combining stage-specific gene inactivation and differential expression analysis with stage-specific GATA4/6 chromatin occupancy maps will be pivotal to elucidate the mechanistic basis for the unique requirement for GATA4/6 in fetal, newborn, and adult heart.

Our studies demonstrate that AAV9:Tnnt2-Cre is a valuable reagent for ablation of floxed genes in cardiomyocytes. AAV9-mediated delivery of Cre recombinase is a highly efficient, cost-effective, and simple method to control the timing and extent of cardiomyocyte-restricted gene knockout. The use of an independently developed AAV9:Tnnt2-Cre for cardiac gene inactivation was recently reported [29]. In this study, the authors demonstrated that AAV9:Tnnt2-Cre efficiently ablated floxed *Srf* in the adult heart. Our study further develops this idea by showing the effectiveness of AAV9:Tnnt2-Cre for neonatal cardiomyocyte gene inactivation and by showing how the temporal control of gene inactivation is critical for interpretation of gene function. Furthermore, by demonstrating how reduced dosage of AAV9:Tnnt2-Cre permits creation of genetic mosaics, which provide critical information about cell autonomous gene function. We also provide addition data from closed chest catheterization showing that AAV9-mediated Cre delivery to the heart has minimal effect on cardiac hemodynamic parameters.

Postnatal gene inactivation with AAV9:Tnnt2-Cre has several significant strengths: (1) AAV9:Tnnt2-Cre is highly portable between mouse lines and research institutions. This significantly reduces time and expense for experiments. (2) The timing of gene inactivation can be precisely controlled. As shown in this study, the timing of gene inactivation can dramatically alter the interpretation of experimental results and their interpretation, suggesting that inactivating the gene of interest at the appropriate developmental stage is critically important. Currently, the most common method for inducible, adult-stage gene inactivation is Myh6-MerCreMer [7]. However, the efficiency of gene inactivation with this reagent is variable. Furthermore, the combination of the MerCreMer fusion protein and tamoxifen triggers fibrosis and ventricular dysfunction, which can confound interpretation of experimental results [9,10,30]. AAV9:Tnnt2-Cre offers an orthogonal, temporally controlled approach, and use of both strategies could help to exclude technical issues related to either experimental strategy alone. (3) AAV9:Tnnt2-Cre dose is easily titrated to achieve mosaic cardiomyocyte knockout. As demonstrated in this study, a mosaic approach opens up new experimental opportunities and also avoids non-cell autonomous, indirect consequences of gene knockout. Mosaic knockout by limited AAV9:Tnnt2-Cre differs from limiting tamoxifen administration to Myh6-MerCreMer, in that in the former each transduced cell expresses robust Cre activity, whereas in the latter many cells possess marginal Cre activity. As a result, we observed high concordance between recombination of 2 *Gata4* alleles, 2 *Gata6* alleles, and the Cre-activated reporter.

Proper controls are needed to most accurately interpret gene inactivation experiments with AAV-Tnnt2-Cre. AAV-induced inflammatory responses are minimal in rodents [12], but still should be excluded as the cause for observed cardiac abnormalities through the use of control AAV (e.g. AAV-luciferase). Treatment of control genotypes with AAV-Tnnt2-Cre further reduces the likelihood of Cre toxicity [31] or potential AAV-triggered host responses. The potential for Cre toxicity increases with long term experiments, and a desirable further refinement would be to develop self-inactivating AAV-Cre to reduce the risk of chronic Cre exposure.

In conclusion, the interpretation of genetic knockout experiments hinges on the timing and extent of gene inactivation. AAV9-mediated Cre delivery is a convenient tool to precisely control these critical experimental parameters for cardiac loss of function studies. Using the strengths of the AAV9:Tnnt2-Cre platform, we defined novel roles of GATA4/6 to regulate cardiomyocyte maturation in the newborn heart, and to maintain diastolic function in the adult heart.

## Supporting Information

S1 Table Ejection fraction (%) in G4G6-fl/fl pups following systemic injection of rAAV9:Tnnt2-Cre (intervention) or rAAV9:Tnnt2-Luc (control). (XLSX)

S2 Table Ejection fraction (%) in adult G4G6-fl/fl mice following systemic injection of rAAV9:Tnnt2-Cre (intervention) or rAAV9:Tnnt2-Luc (cont (XLSX)
